# Effect of heat wave at the initial stage in spark plasma sintering

**DOI:** 10.1186/s40064-016-2344-9

**Published:** 2016-06-22

**Authors:** Long Zhang, Xiaomin Zhang, Zhongxiang Chu, Song Peng, Zimin Yan, Yuan Liang

**Affiliations:** Department of Engineering Mechanics, Chongqing University, Chongqing, 400044 China; Department of Theoretical and Applied Mechanics, Chongqing University of Science and Technology, Chongqing, 401331 China; School of Foreign Language, Chongqing University of Science and Technology, Chongqing, 401331 China

**Keywords:** Field assisted sintering, Spark plasma sintering, Thermo-mechanical coupling, Heat wave, Vacancy concentration difference

## Abstract

Thermal effects are important considerations at the initial stage in spark plasma sintering of non-conductive Al_2_O_3_ powders. The generalized thermo-elastic theory is introduced to describe the influence of the heat transport and thermal focusing caused by thermal wave propagation within a constrained space and transient time. Simulations show that low sintering temperature can realize high local temperature because of the superposition effect of heat waves. Thus, vacancy concentration differences between the sink and the cross section of the particles increase relative to that observed during pressure-less and hot-pressure sintering. Results show that vacancy concentration differences are significantly improved during spark plasma sintering, thereby decreasing the time required for sintering.

## Background

 The field assisted sintering technique (FAST) or spark plasma sintering (SPS) originates from pulsed electrical currents flowing directly through a graphite die or powder materials, the process features high energy requirements (typically a few thousand amperes and several volts), high heating rates (up to 1000 K/min), and high temperature gradient processing (Groza and Zavaliangos 2000; Hungria et al. 2009; Zhang et al. 2014). Although several fundamental investigations on the process have been conducted, knowledge of transient mechanism involving pulse currents and high heat rates under pressure remains lacking because of the complex effects of thermal, electrical, and mechanical processes on mass transport (Antou et al. 2015). For example, in original SPS theory, electrical discharges are believed to exist in the gaps between powder particles, these charges can generate plasma to enhance the thermal diffusion ability of material (Kasperski et al. 2013) and promote sintering (Perera et al. 1998). However, no clear evidence has been presented to demonstrate the occurrence of discharges and the presence of spark plasma during the SPS process (Hulbert et al. 2009). Hulbert used a number of different methods, including in situ atomic emission spectroscopy, direct visual observations, and ultrafast in situ voltage measurements under a variety of SPS conditions to investigate the presence of spark plasma and occurrence of discharge during sintering, thereafter concluding that plasma, sparking, or arcing does not occur during either the initial or final stage of SPS (Hulbert et al. 2009). Tomino measured the current passing through Al_2_O_3_ samples and found a value close to zero (Tomino et al. 1997). On the other hand, Wang et al. (2000) found that compact Al_2_O_3_ powder is denser at the edges than in the middle of the sample at short holding times. Wang et al. (2000) concluded that rapid sintering may be attributed to efficient heat transfer because the graphite mold and punches function as heating elements. Rapid sintering may also be attributed to application of a high heat rate during SPS.

The mechanisms of FAST/SPS are commonly described in terms of mechanical, thermal, and electrical effects. However, in non-conductive Al_2_O_3_ powders, high electric currents flow through the surrounding (graphite) die rather than directly through the sample. Thus, electrical fields cannot be considered in this work. Based on a previous investigation, we suppose the following considerations in Al_2_O_3_ powder: first, mechanical and thermal effects dominate the SPS process. Moreover, the velocity of heat conduction exerts obvious lagging effects on the process, which means the Fourier heat conduction law may not be appropriate for the present situation because it implies thermal propagation velocity is infinite. Since discrete compact particles feature remarkable porous structures or non-homogeneous inner structures (it means the original state), obvious non-Fourier heat conduction characteristics may be observed during transient heat conduction under the condition of an extra-high heating rates and temperature gradients. In this case, the relaxation time is not confined to the molecular or lattice levels and completely differs from the heat transfer mechanism observed metal or non-metal materials (Ignaczak 1989; Tamma and Zhou 1998). The existing experiences show that the relaxation time of microstructural materials or discrete particulate materials may have a magnitude of seconds (Tamma and Zhou 1998; Kaminski 1990; Mitra et al. 1995). The relaxation time of compact Al_2_O_3_ powder (at temperatures of 15–25 °C, the average particle size is 130 nm) ranges from 5 to 45 s, as shown by Roetzel et al. (2003). Although working conditions (e.g., temperature, pressure, etc.) often differ from the test environment, but for the transient behavior at the initial sintering stage instead of the whole densification process, non-Fourier heat conduction is feasible. Therefore, heat transport and thermal focusing will be come out in a constrained space and transient timescale. It may be one of keys to reveal the effects of high local temperature on the neck of non-conductive Al_2_O_3_ powder in the early stages of SPS.

**Fig. 1:**
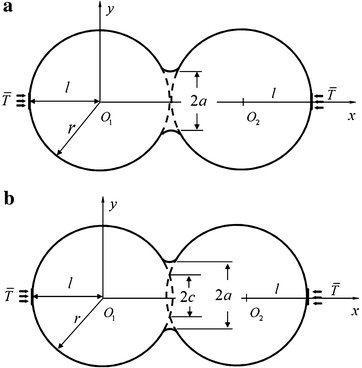
Double equal-sized spherical model. **a** Surface diffusion. **b** Volume diffusion

A double equal-sized spherical model with a particle radius of $r = 0.07\,\upmu{\text{m}}$ is established. Two kinds of diffusion mechanisms (surface diffusion and volume diffusion) are considered. At the initial stage of SPS, surface diffusion causes neck growth but not shrinkage. Thus, no change of the double equal-sized spherical particles in the center-to-center distance can be observed, as shown in Fig. 1a. Under volume diffusion, the neck grows, the center-to-center distance decreases, resulting in shrinkage and densification. This finding is presented in Fig. 1b, where *a* is the neck radius and *c* is half of the length of the cord, in the present case, $c = 0.74a$. The neck growth rate is defined as $X = a/r$. In this paper, we only consider the initial stage of SPS, that is, $X \le 0.3$.

The initial conditions ($t = 0$) are as follows

$$\theta ({\text{x}},{\text{y}},{\text{t}}) = 0,\quad T_{{,{\text{i}}}} ({\text{x}},{\text{y}},{\text{t}}) = 0$$

The boundary conditions are as follows

The step temperature and extra pressure are applied on both ends of the particles, $x = - r,\;3r$:$T = \bar{T}* 1 (t )$, $P = 35\,{\text{MPa}}$. The rest area of particles is adiabatic and stress-free.

The driving force of neck growth is provided by the vacancy concentration gradient. The vacancy concentration difference $\Delta c$ between the sink and the cross section of the particles is described as follows during pressure-less sintering:

1 $$\Delta c = 2c_{0} \cdot \sigma\Omega /kT$$

Here, $\Omega$ is a volume of vacancy, $c_{0}^{{}}$ presents equilibrium concentration of the vacancy of stress free zone, $k$, $T$ are Boltzman constant and absolute temperature, respectively. $\sigma$ is the intrinsic Laplace stress.

On the condition of a high heat rate under pressure, we propose that the vacancy concentration difference of SPS considering the extra applied pressure and thermal stress is shown as follows

2 $${\text{Surface diffusion:}}\;\Delta c = (c_{0}\Omega /a^{2} kT)(2r\gamma + 4Pr^{2} /\pi + a^{2} \Delta \sigma_{T} )$$

3 $${\text{Volume diffusion:}}\;\Delta c = (c_{0}\Omega /a^{2} kT)(4r\gamma + 4Pr^{2} /\pi + a^{2} \Delta \sigma_{T} )$$

here, $\gamma$, $P$ denote the surface energy, and the extra applied pressure. $r$, $a$ are radius of particle and neck. $\sigma_{T}$ is thermal stress caused by the thermo-mechanical interaction. In hot-pressure (HP) sintering, $\sigma_{T} = 0$.

To calculate $\sigma_{T}$, the generalized thermo-elastic equations are introduced (Zhang et al. 2015).

4 $$k^{\prime}\theta_{,ii} = \rho^{\prime}C_{\varepsilon } \left( {\dot{\theta } + \tau_{0} \ddot{\theta }} \right) + \left( {3\lambda + 2\mu } \right)\alpha T_{0} \left( {\dot{\varepsilon }_{kk} + \tau_{0} \ddot{\varepsilon }_{kk} } \right)$$

5 $$\rho\ddot u_i=(\lambda+\mu)u_{j,ij}+\mu u_{i,jj}-(3\lambda+2\mu)\alpha\theta_{,i}$$

here $u_{j}$, $\theta$, $\tau_{0}$ and $\varepsilon_{ij}$ represent the displacement tensor, temperature increment relative to reference temperature $T_{0}$($\theta = T - T_{0}$), relaxation time and the strain tensor, respectively. $k^{\prime}$, $\rho^{\prime}$ and $C_{\varepsilon }$ are the heat conductivity coefficient, density and the specific heat of equal strain. $\lambda$, $\mu$ are lame constants. $\alpha$ represents the coefficient of linear expansion.

The material parameters are described as follows (Wang et al. 2010; Olevsky and Froyen 2009): modulus of elasticity $E = 300\,{\text{GPa}}$, Poisson’s ratio $\nu = 0.22$, $\alpha = 8.0 \times 10^{ - 6} \,\text{K}^{ - 1}$, $\tau = 0.2\,{\text{s}}$, $\rho^{\prime} = 3900\,{\text{kg/m}}^{ 3}$, $k^{\prime} = 27\,{\text{W/(m}}\,{\text{K)}}$, $C_{\varepsilon } = 900\,{\text{J/(kg}}\,{\text{K)}}$, $k = 1.3806505 \times 10^{ - 23} \,{\text{J/K}}$, $\gamma = 1.5\,{\text{J}}\,{\text{m}}^{ - 2}$ and $\Omega = 4.25 \times 10^{ - 29} \,{\text{m}}^{ 3}$.

**Fig. 2:**
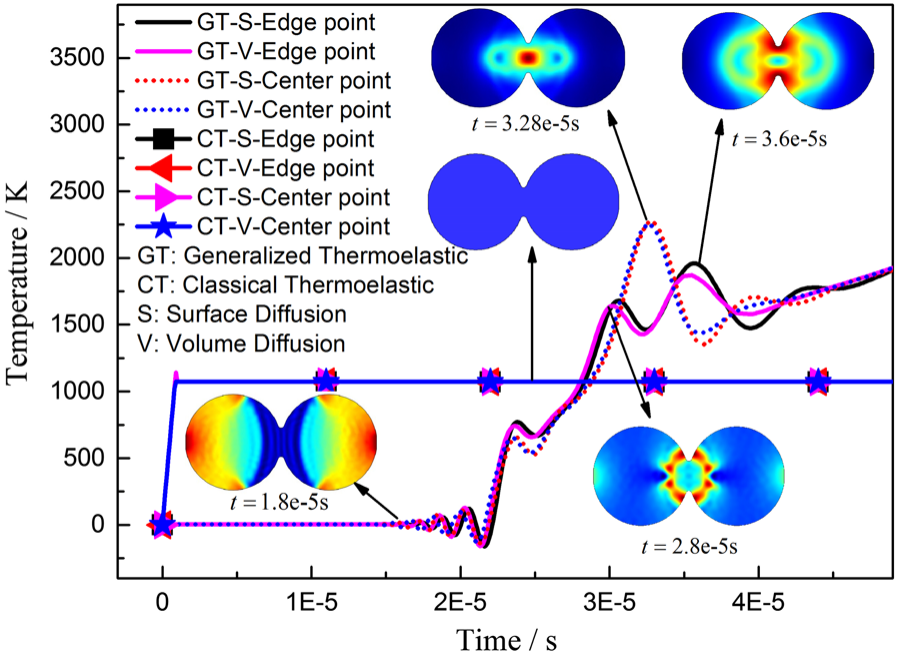
Temperature distribution with the heat wave propagation

Figure 2 clearly demonstrates the thermal focusing effect because of superposition of heat waves under generalized thermo-elastic theory as well as the non-Fourier heat conduction law. It cannot access from the classical Fourier heat conduction law since it implies that thermal diffusion velocity is infinite (the classical Fourier heat conduction law may apply to the conventional pressure-less sintering and hot press sintering). At the center point of the neck, no significant temperature difference was noted between the surface diffusion and volume diffusion mechanisms. However, the difference between these diffusion mechanisms appears at the edge of the neck because shrinkage between two centers of particles can be observed. The superposition effect of heat waves induces a maximum ultimate temperature (about 2242 K), which is much larger than the given temperature (1073 K). It should be notice that the zones close to the neck in both particles will keep at high temperature soon afterwards due to the reflection and secondary superposition of heat wave. However it is just the result of theoretical deduction and it is hard to get the experimental monitoring under the same conditions actually, but we can find some related phenomena in the similar experiments. For non-conductive Al_2_O_3_, Carney and Mah (2008) pointed out the shrinkage began at 988 K (heating at 110 K/min). Xiong and Wang (2008) and Wang and Fu (2002) reported that the center temperature of the sample is higher than the boundary. The biggest temperature difference between the center and the border point is about 220 K (for non-conductive BN powder) and 450 K (for TiB_2_ + BN ceramic composite). Besides Kim and Johnson (1983) put forward a model which requires firing temperatures in the range of 3400–3800 K for 1-μm-radius alumina particles and 5500–7600 K for 10-μm-radius particles. Since the melting point of alumina is 2320 K, such absurdly large numbers clearly indicate the breakdown of conventional sintering mechanism models (Young and McPherson 1989).

**Fig. 3:**
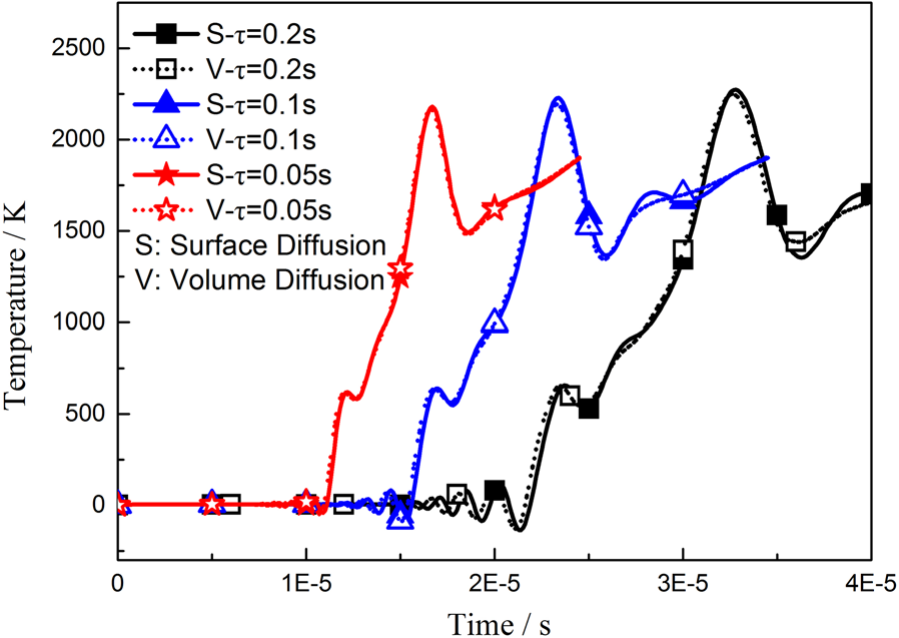
Influence of relaxation time on temperature at center point

The relaxation time is an important parameter used to determine the disciplinarian of propagation time scale and space scale. Figure 3 shows no obvious temperature difference when we take different relaxation times for the same point. In fact, only the arrival times of the wave fronts differ.

**Fig. 4:**
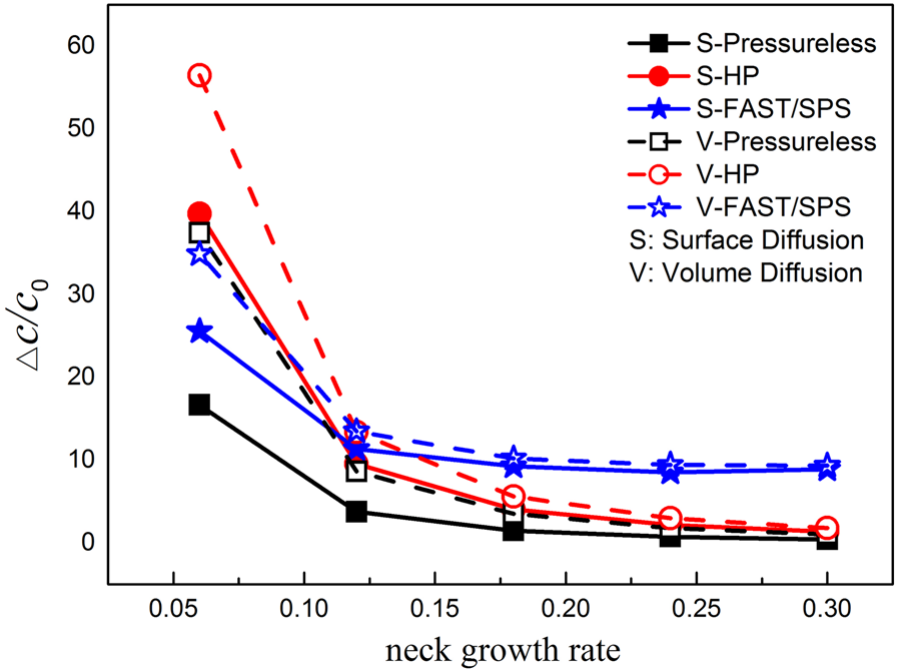
Changes of the concentration difference by neck growing rate in different sintering methods (extra pressure is 35 MPa, the sintering temperature is 1273 K)

Figure 4 shows that the concentration difference decreases as the neck growth rate increases in different sintering methods (e.g., pressure-less, HP and SPS) according to the surface diffusion or volume diffusion mechanism. The magnitude and speed observed are fairly high at the beginning of sintering but decrease thereafter, likely because the effect of stress concentration decreases with increasing curvature of the neck, the corresponding thermal stress changes in the same manner. As the neck grows, the decreasing concentration difference gradually resembles that found during pressure-less sintering and HP. However, in SPS, the concentration difference retains a relatively higher value, that is, about 3–5 times that of the other processes. It will accelerate the sintering proceed in a short time.

## Conclusions

In summary, the generalized thermo-elastic coupling equations were introduced to explain how low sintering temperatures realize high local temperatures in compact Al_2_O_3_ under the condition of an extra-high heat rate and temperature gradient during FAST/SPS. Calculations of the vacancy concentration difference showed that the driving force of sintering during FAST/SPS is indeed much greater than that during conventional pressure-less or HP sintering. In general, the influences of temperature and temperature gradient are collectively belong to the thermal effect in the SPS process, however, for the non-conduction powder compact, the influence of heat wave in the initial stage sintering should not be a ignored factor which have not mentioned before.
